# Parental acceptance and knowledge of varicella vaccination in relation to socioeconomics in Sweden: A cross-sectional study

**DOI:** 10.1371/journal.pone.0256642

**Published:** 2021-10-21

**Authors:** Lisen Arnheim-Dahlström, Natalie Zarabi, Karin Hagen, Goran Bencina

**Affiliations:** 1 Lisen Arnheim-Dahlström Consulting, Stockholm, Sweden; 2 MSD, Stockholm, Sweden; 3 MSD, Madrid, Spain; International Medical University, MALAYSIA

## Abstract

Varicella infection is a highly contagious disease which, whilst mild in most cases, can cause severe complications. Varicella vaccination is available privately in Sweden and is currently being reviewed for inclusion in the Swedish Public Health Agency’s national immunisation program (NIP). A cross-sectional study of parents of Swedish children aged 1–8 years (n = 2212) was conducted to understand parental acceptance, beliefs and knowledge around varicella infection and vaccination. Respondents generally viewed varicella infection as a mild disease, with only a small proportion aware of potential severe complications. While 65% of respondents were aware of the vaccine, only 15% had started the course of vaccination as of February 2019. Further, 43% of parents did not intend to vaccinate, most commonly due to lack of inclusion in the NIP, but also due to perception of mild disease. Nevertheless, if offered within the NIP, 85% of parents would be highly likely to vaccinate their child. A number of statistically significant differences in awareness and behaviours were observed between sociodemographic subgroups. In general, women were more aware of vaccination (72%) compared to men (58%). Among unemployed or respondents with elementary school education, awareness was below 43%, and among respondents with high income the awareness was above 75%. Similarly, among unemployed or respondents with a low income the vaccination rate was as low as 30% compared with at least 57% among respondents with a high income. Respondents from metropolitan areas, those with university degrees and respondents with a higher income were more likely to be aware of the varicella vaccine and to have vaccinated their child. Whilst inclusion in the NIP is clearly the main driver for uptake, these identified knowledge gaps should inform educational efforts to ensure that all parents are informed of the availability and benefits of the varicella vaccine independent of socioeconomic status.

## Introduction

Varicella virus (commonly known as chickenpox) is a highly contagious disease acquired by the majority of children in Western countries, with an annual incidence of approximately one birth cohort [1–3]. Although generally considered a mild disease of children with most cases not requiring medical attention, the risk for severe disease increases with age and is also elevated in the immunosuppressed [2–4].

The Swedish National Immunisation Program (NIP) offers vaccines to all children free of charge, on a voluntary basis. The current program which offers vaccines against 11 pathogens, achieved a vaccine coverage rate (VCR) of 97% among 2-year olds in 2019 [5]. Varicella vaccination is not included in the NIP (as of November 2020), however the Public Health Agency (PHA) is currently reviewing the medical value and cost effectiveness of its inclusion [6]. In the case of a positive recommendation for the inclusion of varicella vaccination in the NIP, a number of factors could influence vaccine uptake. In 2016, the PHA conducted a survey of parents with children aged 0–15 years to assess attitudes towards vaccines in the NIP among the Swedish population [7]. The survey found that most parents (79%) have confidence in the NIP, leading to a high overall VCR [7]. Among parents who had concerns or refused at least one vaccination, the main reasons for doing so were a worry of adverse events, having read or heard negative information as well as lacking reliable information about vaccinations [7]. However, little is known about parent’s perceptions in relation to varicella infection and varicella vaccine acceptability in Sweden. To our knowledge, no study has evaluated knowledge of varicella infection, vaccine acceptability and beliefs in the context of socio-demographics on a national level at the time of writing.

There is a general perception of varicella infection as a mild disease, and as the Swedish NIP is voluntary, this view could influence the VCR if parents choose not to vaccinate their children. The World Health Organization (WHO) highlighted the importance of maintaining a high and sustained VCR for varicella vaccination [3]. Modelling has suggested that in high income countries, a VCR maintained ≥80% is needed to minimise risk of increased morbidity due to a shift in age at infection [3]. Therefore, parental attitudes towards varicella vaccination could play a major role in the success of national immunisation, and so it is crucial to address motivations and potential barriers to uptake. Further, it is important to identify sociodemographic differences to enable focus of education materials on relevant populations. Inclusion in the NIP could have a considerable impact on the incidence of varicella infection, as seen in Germany, where universal vaccination correlated with a reduction in varicella cases by 84%, varicella-associated complications in outpatients by 93%, and varicella infection-associated hospitalisations in children by 60% from 2005 to 2012 [8]. Further, in Finland, varicella vaccination was introduced to the NIP in 2017, with a trend towards reduced incidence of infection and fewer associated healthcare visits already observed [9].

Here we report findings from a cross-sectional study which was performed to 1) identify and understand parental acceptance, beliefs, and knowledge around varicella vaccination in relation to sociodemographic factors and 2) explore participation in the current childhood vaccination program. The focus of this paper is to identify motivations and barriers to vaccination uptake and provide information to guide future educational initiatives.

## Methodology

### Population and study design

The target population of the study were 2000 biological parents or legal guardians of Swedish children aged 1–8 years old (who would be eligible for varicella vaccination NIP if included). Additional eligibility criteria included the ability to read and answer in Swedish, and living in Sweden at the time of responding to the questionnaire. The sample included respondents who had already participated in other surveys, and quotas were applied for gender, age, region and working status at a country level. Respondents of the survey were panellists from a nationally representative web panel, where recruitment was based on random samples. Through their participation in the panel and by conducting surveys, the panellists qualified for rewards such as cinema tickets. Questionnaires were administered from 4^th^ February 2019 to 19^th^ February 2019 via on online platform after obtaining a written informed consent, in line with IRB/ERC requirements. Further, all data were fully anonymised before accessing for analysis.

### Survey questionnaire

A 24-item online questionnaire with closed multiple choice questions and no follow up was used to assess attitudes towards vaccination, attitudes and knowledge about varicella infection and varicella vaccination, and sociodemographic characteristics. Questionnaire topics and key variables are outlined in Table 1; the full questionnaire can be found in S1 Table.

**Table 1:** Survey sections and key variables.

Variable	Respondent characteristics
Section 1: Socioeconomic and sociodemographic characteristics	Age and gender of parent, region, education, religion/philosophical/personal beliefs, monthly household income level, place of birth, nationality, number of children
Section 2: Vaccination status and general vaccination acceptability	If the child is vaccinated according to NIP, reason for not vaccinating according to NIP, religious/philosophic influence on vaccination, knowledge about varicella vaccination, feelings about vaccinating the child for all vaccines included in the NIP, motivators and barriers for varicella vaccination including NIP vaccines
Section 3: Knowledge of varicella vaccination and risk of complications	Varicella burden awareness, perceived severity of varicella infection, benefits of reducing the risk of varicella complications, sources of information of varicella infection, frequency and severity of the disease, target people that can become infected, complications due to varicella infection and ways to avoid being infected

### Sample size and statistical analysis

It was calculated that a sample size of 2000 parents would be sufficient to yield statistically significant results, based on the parental population of children aged 1–8 years old in 2018 in Sweden, and a confidence level of 95% with a margin error of 2%. Pearson’s Chi-squared tests were used to analyse the relationship among variables and assess statistical significance. Odds ratio analysis was then conducted on any variables found to be statistically significant to assess the likelihood of the given outcome. Data are reported as n (%), where n is the number of respondents, and % is the proportion of respondents. 95% confidence intervals are also reported, where appropriate.

The covariate analysis included sociodemographic variables including gender of parent, region, number of children, education, occupation and household income level. All data were analysed using SPSS software.

## Results

A total of 2396 questionnaires were carried out to ensure 2000 answers with near equal representation of each age category of children. Respondents without children were excluded, resulting in a total of 2212 participants. The demographics of the study population are illustrated in Table 2.

**Table 2:** Demographics of respondents (n = 2212).

		Total		Male		Female	
		N	%	N	%	N	%
**Area**	Metropolitan	903	40.8%	445	40.9%	458	40.7%
	Mid-size	1120	50.6%	550	50.6%	570	50.7%
	Rural	189	8.5%	93	8.5%	96	8.5%
**Number of children** *	One	1235	55.8%	595	54.7%	640	56.9%
	Two	863	39.0%	432	39.7%	431	38.3%
	Three or more	114	5.2%	61	5.6%	53	4.7%
	Don’t want to respond	0	0.0%	0	0.0%	0	0.0%
**Year of birth of child** **	2011	260	11.8%	133	12.2%	127	11.3%
	2012	356	16.1%	188	17.3%	168	14.9%
	2013	307	13.9%	161	14.8%	146	13.0%
	2014	279	12.6%	140	12.9%	139	12.4%
	2015	238	10.8%	122	11.2%	116	10.3%
	2016	240	10.8%	108	9.9%	132	11.7%
	2017	251	11.3%	114	10.5%	137	12.2%
	2018	261	11.8%	115	10.6%	146	13.0%
	Don’t want to respond	20	0.9%	7	0.6%	13	1.2%
**Education**	Elementary school	25	1.1%	19	1.7%	6	0.5%
	High school	582	26.3%	354	32.5%	228	20.3%
	Training school	118	5.3%	57	5.2%	61	5.4%
	University	1484	67.1%	657	60.4%	827	73.6%
	Don’t want to respond	3	0.1%	1	0.1%	2	0.2%
**Household income**	150,000 SEK/month or more	25	1.1%	14	1.3%	11	1.0%
	90,000–149,999 SEK/month	251	11.3%	140	12.9%	111	9.9%
	70,000–89,999 SEK/month	606	27.4%	315	29.0%	291	25.9%
	50,000–69,999 SEK/month	635	28.7%	312	28.7%	323	28.7%
	30,000–49,999 SEK/month	441	19.9%	215	19.8%	226	20.1%
	10,000–29,999 SEK/month	120	5.4%	43	4.0%	77	6.9%
	Less than 10,000 SEK/month	8	0.4%	0	0.0%	8	0.7%
	No household income	3	0.1%	1	0.1%	2	0.2%
	Don’t want to respond	123	5.6%	48	4.4%	75	6.7%
**Occupation**	Full time work	1765	79.8%	989	90.9%	776	69.0%
	Part time work	285	12.9%	47	4.3%	238	21.2%
	Unemployed	23	1.0%	9	0.8%	14	1.2%
	Other	139	6.3%	43	4.0%	96	8.5%
**Country of birth of respondent**	Sweden	2117	95.7%	1040	95.6%	1077	95.8%
	Other	95	4.3%	48	4.4%	47	4.2%
**Background**	Both parents born in Sweden	1128	51.0%	540	49.6%	588	52.3%
	One parent born in Sweden	823	37.2%	420	38.6%	403	35.9%
	Both parents born outside of Sweden	261	11.8%	128	11.8%	133	11.8%

* Different respondent base for number of children as based on original sample; n = 2396. Respondents with no children screened out.

** Respondents with several children were asked to account for the child with the most recent birthday when answering the questions.

Among survey participants, gender distribution was even (50.8% female:49.2% male). Metropolitan areas (40.1%) and mid-size cities (50.6%) were well represented, with fewer respondents in rural areas (8.5%), which is representative of the population demographics. Just over half of respondents had one child (52%), and the vast majority were married or co-living with partners (91%). The majority of respondents were university educated (67%), working full time (71%) and had a household income up to 89,999 SEK/month (81%).

### Attitudes towards vaccination

With respect to vaccination in general, 96% of respondents stated that their opinion on vaccination is not influenced by anthroposophy, homeopathy, alternative medicine, or religion. Further, 98% of parents noted that their child has been vaccinated in line with the NIP. Of the respondents who declined vaccines, rotavirus was the most commonly declined vaccine, followed by the MMR vaccine, with the main reasons for declining vaccines stated as the belief that the child doesn’t need it, contraindications, and fear of side effects. Importantly, the low respondent base for those who declined vaccines (n = 39) limits interpretation of these data.

### Perception of varicella infection severity

**Fig 1:**
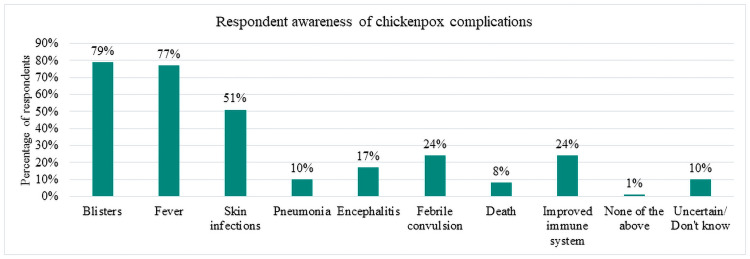
Bar chart showing percentage of overall respondents aware of different varicella complications (n = 2212).

Regarding varicella infection, respondents generally viewed it as a mild disease relative to other infectious diseases. Among the pre-defined options, meningococcus, cervical cancer/human papillomavirus (HPV), polio and tetanus were considered the most severe conditions, whilst varicella infection and seasonal flu were perceived to be the least severe. Only 17.0% (95% CI:15.5%– 18.6%) of respondents agreed with the statement ‘Chickenpox is a severe disease’ and 8% agreed that ‘It is likely that a child suffers from complications due to chickenpox’. Household income was the only variable for which statistically significant differences in response were seen for the perception of varicella infection as a severe disease (95% CI: 15.5% to 18.6%). Regarding varicella complications (Fig 1), there was a greater awareness of less severe complications, such as blisters (79% awareness) and fever (77% awareness), whilst only 17% were aware that varicella infection could cause encephalitis, and 10% were aware of the potential for pneumonia. Further, as many as 24.4% (95% CI:22.6%– 26.2%) believed that varicella infection leads to an improved immune system. With respect to the perception that the infection improves the immune system, few differences were seen between sociodemographic groups, however a higher share of respondents were training school graduates compared to high school graduates (90% confidence level). Overall, these findings confirm varicella infection being generally considered mild with parents having limited awareness of the potential severity of infection, further that this is largely independent of sociodemographic variables.

### Awareness and attitudes towards varicella vaccination

Overall, 64.7% (95% CI:62.7% - 66.7%) of respondents were aware of the varicella vaccine (S1 Fig). However, in this case a number of statistically significant differences in awareness were seen between different sociodemographic groups (Table 3). Awareness was higher in women than men (OR:1.84 [95% CI:1.54–2.20]), and those from metropolitan areas as compared to mid-size (OR:0.65 [95% CI:0.54–0.78]) and rural areas (OR:0.48 [95% CI:0.35–0.66]). Further, respondents with university degrees, parents of two children, those with a higher household income, and respondents in part-time work were more likely to be aware of the vaccine (Table 3). Awareness was lower in unemployed than fulltime workers (OR: 0.44 [95% CI:0.19–1.01]), highlighting the need for targeted educational efforts.

**Table 3:** Odds ratio analysis of awareness of varicella vaccine and vaccination status for all significant variables based on Chi-squared test.

		Total number of respondents	Aware of varicella vaccine	Odds ratio (95% CI)	Total number of respondents	Have vaccinated or intend to vaccinate	Odds ratio (95% CI)
		N(%)	N (%)		N* (%)	N (%)	
**Gender**	Male	1088 (49%)	628 (57.7%)	**Reference**	561 (49%)	241 (43.0%)	**Reference**
	Female	1124 (51%)	804 (71.5%)	1.84 (1.54–2.20)	583 (51%)	234 (40.1%)	-
**Area**	Metropolitan	903 (41%)	642 (71.1%)	**Reference**	447 (39%)	216 (48.3%)	**Reference**
	Mid-size	1120 (51%)	688 (61.4%)	0.65 (0.54–0.78)	598 (52%)	226 (37.8%)	0.65 (0.51–0.83)
	Rural	189 (9%)	102 (54.0%)	0.48 (0.35–0.66)	99 (9%)	33 (33.3%)	0.54 (0.34–0.85)
**Number of children**	1	1235 (52%)	758 (61.4%)	**Reference**	664 (58%)	268 (40.4%)	**Reference**
	2	863 (36%)	596 (69.1%)	1.41 (1.17–1.69)	431 (38%)	180 (41.8%)	-
	3+	114 (5%)	78 (68.4%)	1.36 (0.90–2.06)	49 (4%)	27 (55.1%)	-
	Don’t want to respond	184 (8%)	0 (0.0%)	-	0 (0%)	0 (0.0%)	-
**Education**	Elementary school **	25 (1%)	9 (36.0%)	-	14 (1%)	8 (57.1%)	-
	High school	582 (26%)	326 (56.0%)	**Reference**	291 (25%)	95 (32.6%)	**Reference**
	Training school	118 (5%)	69 (58.5%)	1.11 (0.74–1.65)	60 (5%)	17 (28.3%)	0.82 (0.44–1.51)
	University	1484 (67%)	1026 (69.1%)	1.76 (1.44–2.14)	777 (68%)	355 (45.7%)	1.74 (1.31–2.30)
	Don’t want to respond	3 (0%)	2 (66.7%)	-	2 (0%)	0 (0.0%)	-
**Household income**	90,000+ SEK/month	276 (12%)	215 (77.9%)	**Reference**	126 (11%)	72 (57.1%)	**Reference**
	70,000–89,999 SEK/month	606 (27%)	407 (67.2%)	0.58 (0.42–0.81)	297 (26%)	139 (46.8%)	0.66 (0.43–1.00)
	50,000–69,999 SEK/month	635 (29%)	396 (62.4%)	0.47 (0.34–0.65)	332 (29%)	138 (41.6%)	0.53 (0.35–0.81)
	30,000–49,999 SEK/month	441 (20%)	259 (58.7%)	0.40 (0.29–0.57)	242 (21%)	79 (32.6%)	0.36 (0.23–0.57)
	<29,999 SEK/month	131 (6%)	77 (58.8%)	0.41 (0.26–0.63)	79 (7%)	22 (27.8%)	0.29 (0.16–0.53)
	Don’t want to respond	123 (6%)	78 (63.4%)	-	68 (6%)	25 (36.8%)	-
**Occupation**	Full time work	1560 (71%)	1122 (63.6%)	**Reference**	895 (78%)	392 (43.8%)	**Reference**
	Part time work	285 (13%)	202 (70.9%)	1.40 (1.06–1.83)	165 (14%)	51 (30.9%)	0.57 (0.40–0.82)
	Unemployed	23 (1%)	10 (43.5%)	0.44 (0.19–1.01)	15 (1%)	4 (26.7%)	0.47 (0.15–1.48)
	Other	344 (15%)	98 (70.5%)	1.37 (0.94–2.00)	69 (6%)	28 (40.6%)	0.88 (0.53–1.44)

Note: only variables for which Chi^2^ test found statistical significance were included in the odds ratio analysis. Respondent bases for the two questions differed, as all respondents were asked about awareness of the vaccine, but only those who reported that their child had not had chickenpox answered to whether they had vaccinated or intended to vaccinate.

*Total number of respondents that answered whether they had vaccinated or intent to vaccinate.

** Elementary school was not included in the odds ratio analysis due to the low respondent base.

In determining which platforms could be used for educational initiatives, it was noted that across all respondents, friends and family were the most common source of information on the vaccine (34%). However, men were more likely to receive information from child health care (34%) than family and friends (29%). Internet searches (17%) and media (14%) were also commonly reported sources of information on the vaccine across all respondents.

Among the study sample, 48% of children had been infected with varicella, most commonly with a disease duration of 1–2 weeks. Among children who had not had varicella infection, 15% had completed or started the course of vaccination, a further 26% of parents intended to vaccinate their children. However, 43% stated that they did not intend to vaccinate against varicella. Among the 41.5% of respondents who had vaccinated their child or intended to vaccinate, statistically significant differences were seen depending on the parent’s occupation, with full-time workers most likely to vaccinate. Respondents with a higher household income and those from metropolitan areas were also significantly more likely to vaccinate (S2 Fig; Table 3). Parents with university degrees were more likely to have vaccinated or intended to vaccinate than high school graduates (OR:1.74 [95% CI:1.31–2.30]), whilst training school graduates were less likely to have vaccinated compared to university graduates (OR:0.82 [95% CI:0.44–1.51]). Though a higher proportion of those who had only attended elementary school had vaccinated or intended to vaccinate their child, this difference was not found to be significant.

**Fig 2:**
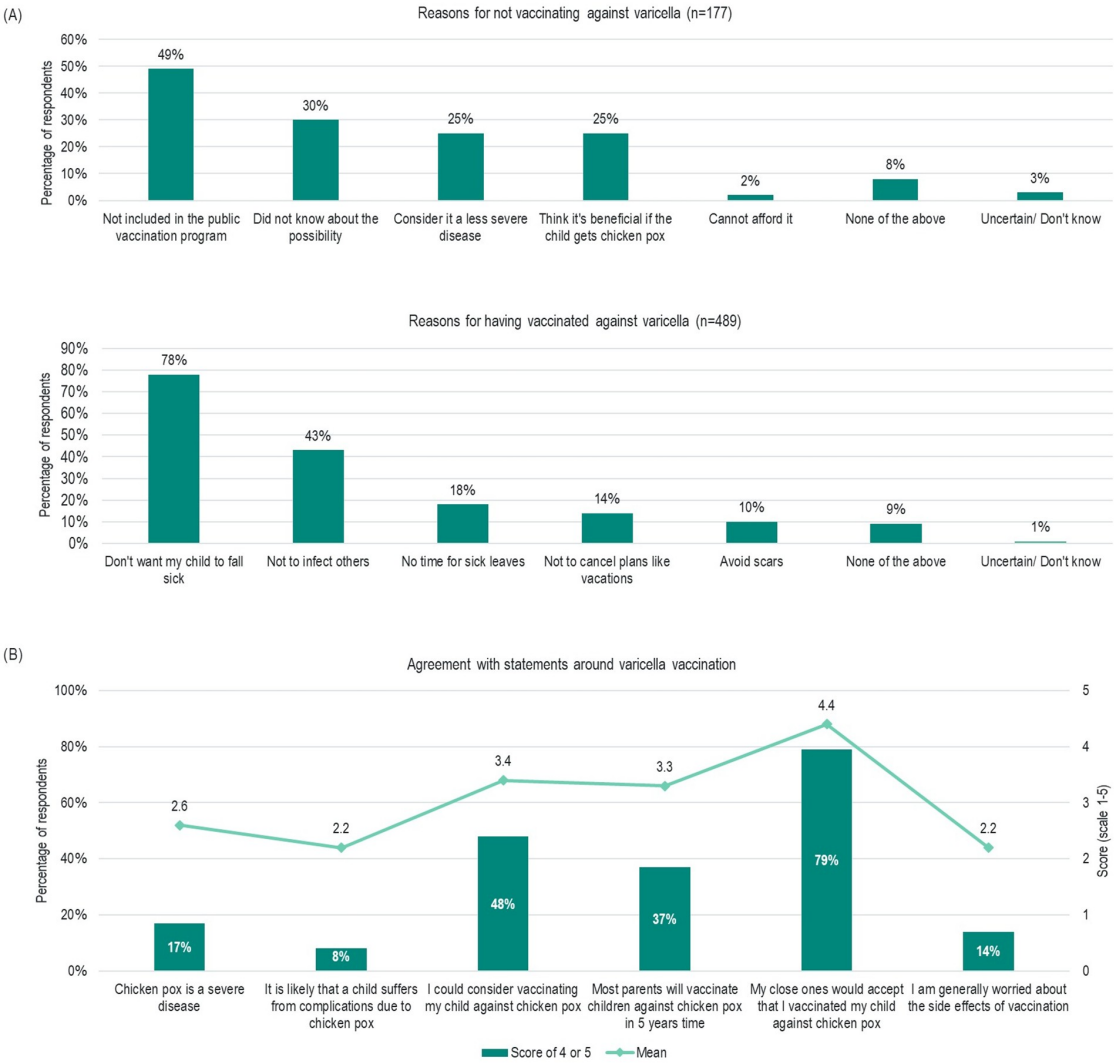
Perceptions of varicella vaccination. (A) Bar charts showing reasons for parents not vaccinating child against varicella infection (n = 177) and reasons for having vaccinated against varicella infection (n = 489); (B) Graph showing respondent agreement with statements around varicella vaccination, from total study sample (n = 2212). Respondents were asked to rate how strongly they agree or disagree with each statement on a scale of 1 to 5, where 5 is strongly agree. Mean scores and the percentage of respondents rating each statement as 4 or 5 are reported.

The most commonly reported reasons for not intending to vaccinate against varicella were its lack of inclusion in the NIP (49%), lack of awareness of the possibility of vaccinating (30%), the perception of varicella infection as a mild disease (25%) and that children could benefit from having the infection (25%) (Fig 2). Meanwhile those who had already vaccinated their children cited concern for the wellbeing of their own child (78%) and for the wider population (43%) as the main reasons for doing so. Practical concerns around sick leave from work, cancelling vacations, and aesthetic reasons such as avoiding scars, were less likely to be reported as reasons for vaccinating (Fig 2). Overall, these findings provide important insight into how best to engage parents and encourage enhanced vaccination uptake.

In line with this, attitudes towards a varicella vaccine were generally positive. 48% of parents would consider vaccination, 37% agreed with the statement that ‘Most parents will vaccinate children against chickenpox in 5 years’ time, and 79% with the statement ‘My close ones would accept that I vaccinated my child against chickenpox’ (Fig 2). Further, in focus on varicella vaccination, 14% of respondents agreed with the statement ‘I am generally worried about the side effects of vaccination.’ Ultimately, if offered within the NIP, 85% of parents would be highly likely to vaccinate their child, with a mean likelihood of 4.5 (scale 1–5; 1 = not likely at all, 5 = very likely).

## Discussion and conclusions

To our knowledge, this is the first study evaluating knowledge of varicella infection, and acceptance and awareness of varicella vaccination, in relation to sociodemographics in Sweden on a national level. Overall, the results showed limited awareness of the varicella vaccine, as well as poor knowledge of the potential severity of the infection. This highlights the need for educational initiatives to raise this level of awareness among Swedish parents, particularly among certain populations.

Despite the potential for severe complications of varicella infection, including neurological involvement and secondary bacterial infections [1–3], varicella infection was not generally perceived as a severe disease by survey respondents, with only a small proportion of parents aware of the potential severe complications. Indeed, as seen in a study by van Lier et al. in the Netherlands, flu and varicella infection were perceived as the two least severe among a list of infectious diseases [10]. With respect to knowledge of varicella vaccination, only 65% of Swedish parents were aware that vaccination was possible, and only 15% of parents had vaccinated their child against varicella. The low proportion of children who had been vaccinated against varicella infection may not be surprising given its lack of inclusion in the NIP. However, this vaccination rate, and awareness of varicella vaccination, was lower than that seen in other countries. Of note, in Hungary, Huber et al. found that 53.3% of parents had vaccinated at least one child against varicella infection, whilst in Italy, Vezzosi et al. found 82.6% awareness of varicella vaccination but a vaccination rate of only 38.4%, with both these countries having availability of varicella vaccination, but not free of charge within the NIP at the time of the study [11, 12]. In Hong Kong, Tam et al. found a much higher vaccination rate, with 69% of respondents having vaccinated their child against varicella infection despite its absence from the NIP at the time of the study [13]. Importantly, any comparisons between countries should be taken with caution, as differences may be attributed to study design and inclusion criteria, rather than differences in beliefs and attitudes on a population level.

Despite the low rate of varicella vaccination in Sweden, attitudes towards a vaccine were positive, with 85% of parents highly likely to vaccinate their child if varicella vaccination was included in the NIP. Importantly, this was above the 80% coverage rate recommended by WHO to avoid a shift in the age distribution of the infection [3].

A number of statistically significant differences in attitudes were observed between sociodemographic subgroups, though overall, differences between groups were relatively small. In particular, variations were identified in relation to awareness of the vaccine, where women, respondents with university degrees, those with a higher income, and part-time workers were more likely to be aware of it. Meanwhile, respondents from metropolitan areas were more likely to be aware of the varicella vaccine than those from mid-sized or rural areas, and respondents with two children were more likely to be aware than those with only one child. Looking at the proportion of respondents who had vaccinated their child against varicella infection, or intended to do so, similar variations were seen. Again, respondents from metropolitan areas were most likely to have vaccinated, alongside those with university degrees and respondents with a higher income. However, in this instance full-time workers were more likely to have had their child vaccinated than part-time workers or unemployed respondents. These sociodemographic variations are in line with differences seen in other countries. In Hong Kong, Hungary, and Italy, respondents with a higher level of education were also more likely to have vaccinated their child against varicella infection, or be aware of the vaccination [11–13]. This may be explained by the fact that parents with a higher education background may have better access to healthcare information. Ensuring access to healthcare information for lesser educated parents will be key in raising awareness and ensuring uptake if varicella vaccination is included in the Swedish NIP. Interestingly, the opposite trend was seen in France and the Netherlands, where more educated respondents were less likely to have their children vaccinated [14, 15]. The authors of the French study rationalised this as a greater commitment of more highly educated individuals to make health-related decisions combined with a lower trust in authorities [14], while the study in the Netherlands highlighted a perceived low severity of the disease [15]. As discussed previously, this was also a general perception across respondents of this study. In Hungary, respondents living in the capital city were more likely to have vaccinated, in line with the greater awareness and vaccination in metropolitan areas of Sweden [11]. Again, respondents living in more remote, rural areas may have less exposure to healthcare information, so this population may represent a key target for education. Further, given that respondents with a lower household income were found to be less likely to have vaccinated their child against varicella infection, it could be expected that vaccine coverage in this population would increase considerably if the vaccine becomes freely available within the NIP. Indeed, this is corroborated by the finding that inclusion in the NIP would be a key driver of vaccination in the total Swedish cohort. Across all respondents, family and friends were the main source of information on the vaccine, and so word of mouth will be a key driver of awareness should this vaccination be included in the NIP. Importantly, child healthcare and internet searches were common sources of information, and so should be considered as key platforms for messaging to increase awareness.

Looking more broadly at vaccination, in alignment with previous reports, the study found that the vast majority of children in Sweden (98%) have been vaccinated in accordance with the NIP. Further, very few parents were influenced by alternative, anthroposophical or homeopathic medicine, with a generally low fear of side effects from vaccines. However, as this study was conducted in February 2019, it remains to be seen whether the COVID-19 pandemic will have had any impact on parental attitudes towards vaccination going forward.

There are a number of limitations with this study. Firstly, the study design does not enable a causal relationship between perceptions and sociodemographic background to be seen, but correlations can be observed. In addition, not all potential confounding factors could be controlled for in the analysis. As respondents were required to be able to read and answer in Swedish, the attitudes of non-Swedish speaking immigrants were not captured. The profession of the respondent was also not captured, as such some professions, e.g. related to healthcare, may have had the potential to influence data. Further, questions captured the duration of varicella sickness but not the severity, the past experiences of the respondent relating to varicella infection were also not recorded, both of which could have impacted the responses given. Finally, information reported by parents is subjective and could be affected by social desirability, imprecision, or mistakes.

Nevertheless, a clear need exists for education around the potential for varicella complications, and availability of a vaccine. It is important that parents appreciate the potential for severe complications to ensure high uptake, in line with WHO recommendations to maintain a high VCR. An appreciation of the variation in sociodemographic factors and the favoured information channels should help to guide educational efforts. Such efforts should focus on the lesser known varicella complications to combat the widespread perception of varicella infection as a mild disease relative to other infectious diseases.

In conclusion, this study found that a majority of respondents would most likely vaccinate their children if the varicella vaccine was offered within the NIP, with the main reason for parents having not yet vaccinated their children being its current lack of inclusion. Inclusion in the NIP would clearly be a key driver for increasing varicella vaccination rates, which should be supported by educational efforts to ensure parents are informed of the availability and benefits of the vaccine.

## Supporting information

S1 Table Full questionnaire used with respondents. (DOCX)

S1 Fig Awareness of varicella vaccine across total study. Bar chart showing percentage of respondents aware of the potential to vaccinate against varicella infection, stratified by respondent gender, area, number of children, age of child, education, income and occupation (n = 2212).
(TIF)

S2 Fig Child varicella vaccination status by respondent demographics, among respondents reporting that their child has not had chickenpox (n = 1144). (TIF)
