# Supplementary material for: Parental acceptance and knowledge of varicella vaccination in relation to socioeconomics in Sweden: A cross-sectional study
Source: PLoS One. 2021 Oct 21;16(10):e0256642. doi: 10.1371/journal.pone.0256642 (PMC8530319; doi:10.1371/journal.pone.0256642)
Supplement: S1 Table — (DOCX) [file pone.0256642.s001.docx]

**Supplementary Table 1. Full questionnaire used with respondents**

| **Question** | **Options** |
| --- | --- |
| 1. Are you a parent or legal guardian of one or more children aged between 1 and 8? | 1. Yes, one 2. Yes, two 3. Yes three or more 4. No (conclude) 5. Do not want to specify (conclude) |
| 2. If you have several children of this age, please indicate for the youngest child. What is the year of birth of the child about whom you are answering? | 1. 2018 2. 2017 3. 2016 4. 2015 5. 2014 6. 2013 7. 2012 8. 2011 9. Do not want to specify |
| 3. Which is your highest completed educational level? | 1. Elementary school or equivalent 2. Upper secondary school 3. Vocational school 4. College of higher education/university 5. Do not want to specify |
| 4. What is the combined income for the household? | 1. Have no income 2. Less than 10,000 SEK/month 3. 10,000-29,999 SEK/month 4. 30,000-49,999 SEK/month 5. 50,000-69,999 SEK/month 6. 70,000-89,999 SEK/month 7. 90,000-149,999 SEK/month 8. 150,000 SEK/month or more 9. Do not want to specify |
| 5. What is your main employment? | 1. Full-time work 2. Part-time work 3. Self-employed 4. Student 5. On parental leave 6. Retired 7. On long-term sick leave 8. Unemployed 9. Other 10. Do not want to specify |
| 6. What is your civil status? | 1. Single 2. Married/cohabiting 3. Divorced/separated 4. Do not want to specify |
| 7. Where were you born? | 1. Sweden 2. Nordics (except Sweden) 3. Europe (except Nordics) 4. Asia (except Middle East) 5. Middle East 6. Africa 7. North America 8. South America 9. Rest of the world |
| 8. Do you have at least one parent who was born in: | 1. Sweden 2. Nordics (except Sweden) 3. Europe (except Nordics) 4. Asia (except Middle East) 5. Middle East 6. Africa 7. North America 8. South America 9. Rest of the world 10. No, both parents were born in Sweden |
| 9. Is your opinion of vaccination affected by any of the following? Several answer options are possible. | 1. Anthroposophical philosophy 2. Homeopathic philosophy 3. Alternative medicine 4. Religion 5. None of the above 6. Do not want to specify |
| 10. Has your child been vaccinated in accordance with the child vaccination programme offered through a child healthcare centre and/or school? | 1. Yes 2. No, I have declined one vaccine 3. No, I have declined more than one vaccine 4. Not sure, don’t know |
| FILTER: If NO to Q10   11. Which vaccines have your previously declined? Do not choose the Rotavirus option (a) if your child has not been offered the vaccine at a child healthcare centre. | 1. Rotavirus infection 2. Diphtheria, tetanus, whooping cough and polio 3. Hib (Haemophilus influenzae type b) 4. Streptococcus pneumoniae 5. Measles, mumps, rubella |
| FILTER: if NO to Q10  12. Why did you refuse one or more of the vaccinations in the child vaccination programme? | 1. Don't think my child needs the vaccination 2. Religious reasons 3. Afraid of side effects 4. Allergy 5. Medically contraindicated 6. Not sure, don’t know |
| 13. Are you aware that one can be vaccinated against chickenpox? | 1. Yes 2. No 3. Not sure, don’t know |
| FILTER: if YES to Q13  14. Where did you get the information about chickenpox vaccine? | 1. Child healthcare centre 2. GP 3. Vaccination centre 4. Relative/friend 5. Social media, e.g. Facebook 6. Searched the internet 7. Media 8. School healthcare service 9. Nursery school 10. None of the above 11. Not sure, don’t know |
| 15. How serious do you think the following illnesses are? Scale 1-5; 1=not serious at all, 5=very serious | 1. Diphtheria 2. Pertussis (whooping cough) 3. Tetanus 4. Polio 5. Chickenpox 6. Pneumococcal infection (respiratory infection) 7. Cervical cancer (HPV) 8. Hepatitis B 9. Mumps 10. Measles 11. Rubella 12. Rotavirus infection 13. Meningococcal infection (meningitis) 14. Seasonal influenza |
| 16. To what extent do you agree with the following assertions? Scale 1-5; 1=don’t agree at all, 5=totally agree | 1. Chickenpox is a serious illness 2. It is likely that children will get complications as a result of chickenpox 3. I can imagine having my children vaccinated against chickenpox 4. Most parents will choose to have their children vaccinated against chickenpox within 5 years 5. Most people close to me (family/friends) would accept my having my child vaccinated against chickenpox 6. I’m generally worried about side effects/complications of vaccination |
| 17. What complications do you think chickenpox can lead to? | 1. Blisters 2. Fever 3. Skin infection 4. Pneumonia 5. Encephalitis 6. Febrile convulsions 7. Death 8. Strengthened immunological defence 9. None of the above 10. Not sure, don’t know |
| 18. Has your child had chickenpox? | 1. Yes 2. No 3. Not sure, don’t know |
| FILTER: if YES to Q18  19. How long was your child sick with chickenpox? From the first day of sickness to completely healthy | 1. Less than a week 2. 1-2 weeks 3. 2-3 weeks 4. 3 weeks or more 5. Not sure, don’t know |
| FILTER: if NO or DON’T KNOW to Q18  20. Have you had your child vaccinated against chickenpox? | 1. Yes, with one dose of vaccine 2. Yes, with two doses of vaccine 3. No, but I’m planning on vaccination 4. No, and I’m not considering vaccination 5. Not sure, don’t know |
| FILTER: if NO to Q20  21. What is the reason for your not having had your child vaccinated against chickenpox or your not considering this? | 1. I was not aware of vaccination against chickenpox 2. I cannot afford it 3. Chickenpox is a harmless disease 4. I think it is good if my child to be infected with chickenpox 5. Chickenpox vaccination is not included in the vaccination program 6. None of the above 7. Do not know |
| FILTER: if NO to Q20  22. How likely is it that you would have your child vaccinated if vaccination against chickenpox were included in the national child vaccination programme? | Scale 1-5; 1=not likely at all, 5=highly likely |
| FILTER: if YES to Q20  23. Why have you had your child vaccinated against chickenpox? | 1. So my child won’t get ill 2. To avoid scars 3. Can’t take time off to care for my child 4. To avoid having to cancel family plans such as holidays 5. So as not to infect others 6. None of the above 7. Not sure, don’t know |

Questions were provided to biological parents or legal guardians of Swedish children aged 1-8 years old (who would be eligible for varicella vaccination NIP if included). The 24-item online questionnaire was used to assess attitudes towards vaccination, attitudes and knowledge about varicella infection and varicella vaccination, and sociodemographic characteristics.
