# Supplementary figures and images for: Parental acceptance and knowledge of varicella vaccination in relation to socioeconomics in Sweden: A cross-sectional study
Source: PLoS One. 2021 Oct 21;16(10):e0256642. doi: 10.1371/journal.pone.0256642 (PMC8530319; doi:10.1371/journal.pone.0256642)

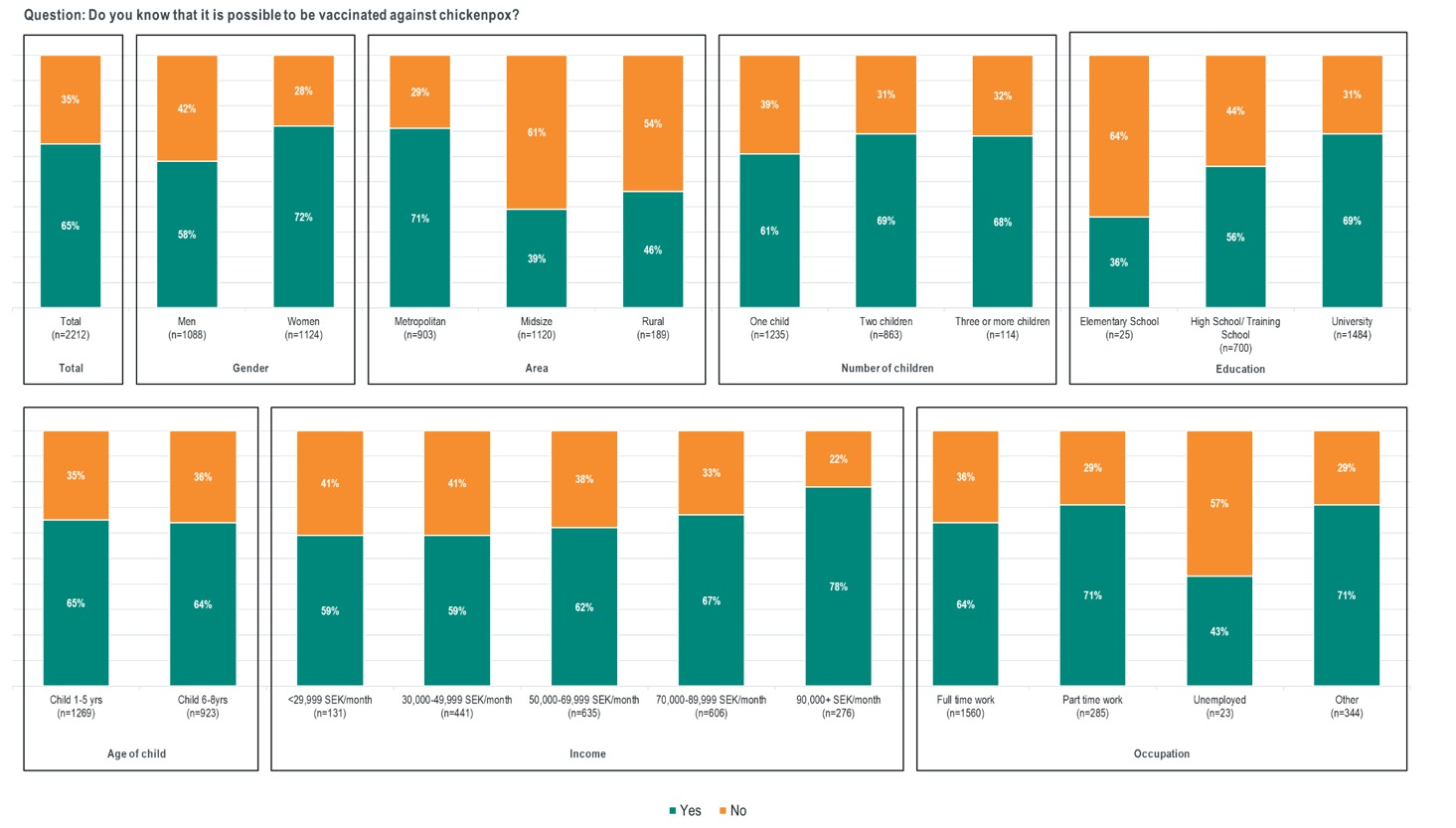

Supplement: S1 Fig — Bar chart showing percentage of respondents aware of the potential to vaccinate against varicella infection, stratified by respondent gender, area, number of children, age of child, education, income and occupation (n = 2212). (TIF) [file pone.0256642.s002.tif]

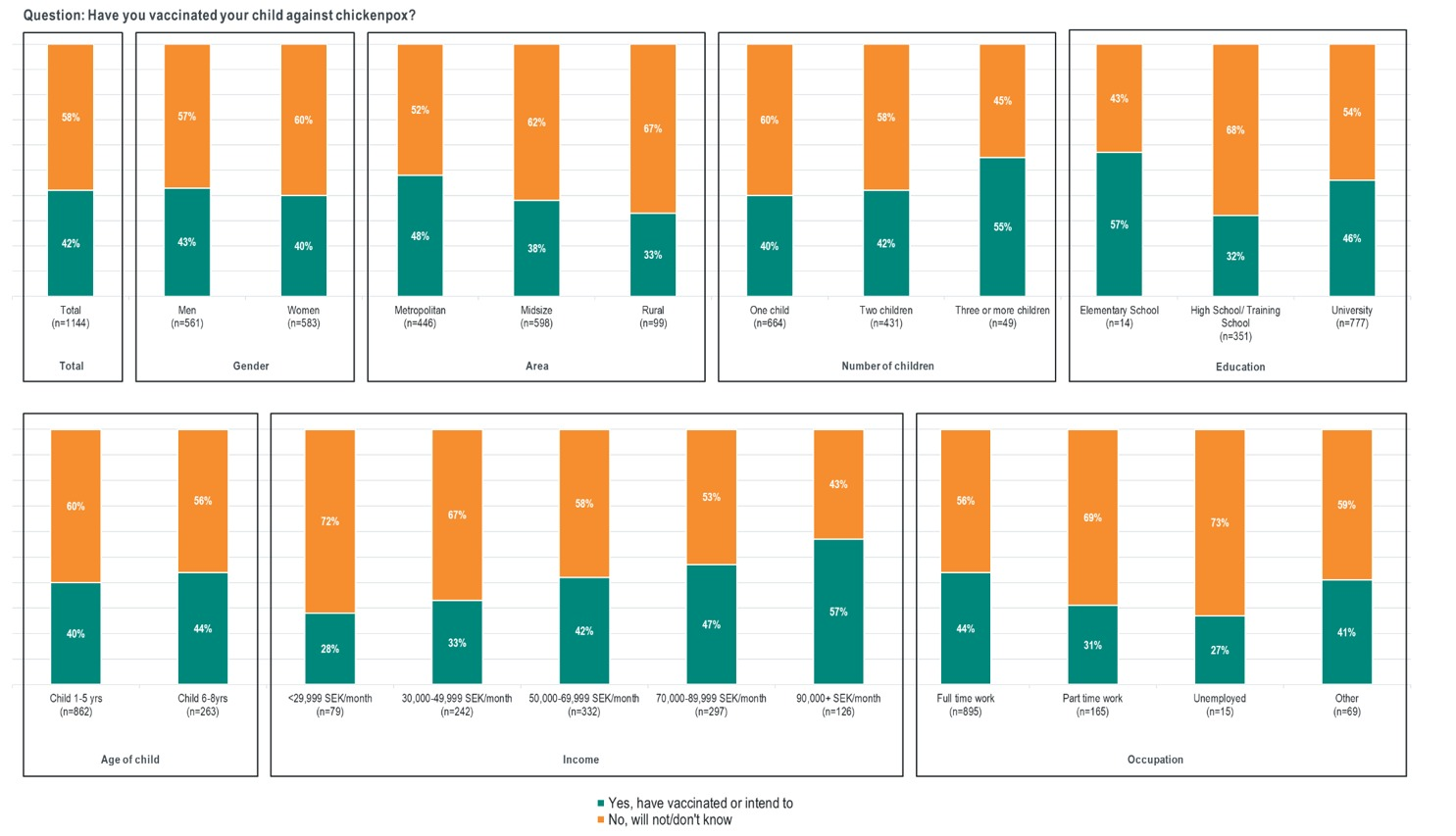

Supplement: S2 Fig — (TIF) [file pone.0256642.s003.tif]
